# Debittering of Protein Hydrolysates by *Lactobacillus* LBL-4 Aminopeptidase

**DOI:** 10.4061/2011/538676

**Published:** 2011-08-24

**Authors:** Bozhidar Tchorbanov, Margarita Marinova, Lydia Grozeva

**Affiliations:** Institute of Organic Chemistry with Centre of Phytochemistry, Bulgarian Academy of Sciences, Acad. G. Bonchev Str. 9, 1113 Sofia, Bulgaria

## Abstract

Yoghurt strain *Lactobacillus* LBL-4 cultivated for 8–10 h at pH ~6.0 was investigated as a considerable food-grade source of intracellular aminopeptidase. Cell-free extract manifesting >200 AP U/l was obtained from cells harvested from 1 L culture media. Subtilisin-induced hydrolysates of casein, soybean isolate, and *Scenedesmus* cell protein with degree of hydrolysis 20–22% incubated at 45°C for 10 h by 10 AP U/g peptides caused an enlarging of DH up to 40–42%, 46–48%, and 38–40% respectively. The DH increased rapidly during the first 4 h, but gel chromatography studies on BioGel P-2 showed significant changes occurred during 4–10 h of enzyme action when the DH increased gradually. After the digestion, the remained AP activity can be recovered by ultrafiltration (yield 40–50%). *Scenedesmus* protein hydrolysate with DH 20% was inoculated by *Lactobacillus* LBL-4 cells, and after 72 h cultivation the DH reached 32%. The protein hydrolysates (DH above 40%) obtained from casein and soybean isolate (high *Q* value) demonstrated a negligible bitterness while *Scenedesmus* protein hydrolysates (low *Q* value) after both treatments were free of bitterness.

## 1. Introduction

During the enzymatic hydrolysis of proteins (especially with high *Q* value [1]), bitter-taste peptides are released limiting their application in food processing. Methods for elimination of bitter peptides are known, but the procedures cause a significant loss of essential amino acids [2, 3]. Exopeptidases from different sources have been applied for additional hydrolysis of protein hydrolysates reducing the bitter taste. Kidney's tissue aminopeptidase [4] as well as pancreatic protease preparations [5] have been involved in the debittering procedures of different originate peptides. Enzymes from plant origin have been successfully used for this purpose [6–8]. The bitterness of casein hydrolysate as well as of soybean protein hydrolysate has been removed at low cost with incubation by cell wall *Saccharomyces* carboxypeptidases [9]. Similar effect has been observed during the additional hydrolysis of peptides catalyzing by fungal protease preparation [10]. A number of commercial microbial aminopeptidases have been in use for preparation of protein hydrolysates lacking bitter taste [11–14]. 

Special attention was given to aminopeptidases hydrolyzing bitter peptides and liberating aromatic amino acids, which are important precursors of aroma compounds [15–17]. Enzymes hydrolyzing single Pro or pairs of Pro residues in bitter peptides are of particular interest [17–20]. Because of its unusual structure, the proline residue is resistant to enzymatic hydrolysis by exopeptidases and oftentimes limits the depth of hydrolysis that can be achieved. The use of proline-specific peptidases together with aminopeptidases of broad specificity has been especially successful in the food industry [21]. 

A basic review on bitterness in cheese due to the partial casein hydrolysis was published in 1996 [22]. The lactic acid bacteria expressing specific X-prolyl dipeptidylpeptidase is also being explored for debittering of tryptic digests and enzyme-modified cheese [23]. A similar approach has also been used for the generation of bioactive peptides with antihypertensive, immunomodulatory, and antimicrobial properties [24].

Exopeptidase activities have been detected in the number of lactic acid bacteria, but their role has been described in respect mainly to the dairy processes [24, 25]. *Lactobacillus* LBL-4 strain from yoghurt starters was reported as a source of intracellular AP activity [26]. The aim of the present work is to study: (i) *Lactobacillus *LBL-4 as a source of AP activity depending on the fermentation conditions, (ii) the additional cleavage and corresponding debittering of hydrolysates from casein, soybean (high *Q* value), as well as *Scenedesmus* cell protein (low *Q* value) *in vitro *by cell-free AP extract, (iii) hydrolysis *in vivo* during the cultivation of the strain LBL-4 on a medium based on the protein hydrolysate from *Scenedesmus* cells.

## 2. Experimental Procedures

### 2.1. Aminopeptidase Extract

The strain *Lactobacillus* LBL-4 was obtained from the ELBY Engineering Lactic Acid Bacteria Collection, Sofia, Bulgaria. A 10% (v/v) inoculum of the working culture LBL-4 was added to a medium based on hydrolyzed milk proteins [27] in a fermenter LKB 1601 Ultroferm, and temperature was maintained at 45°C for 14 h. 

The cultivation was conducted using two different ways—at natural pH decrease through the lactic acid fermentation as well as at pH 5.9  ±  0.1 maintained by automatic addition of neutralizer containing 20% Na_2_CO_3_ in 20% NH_4_OH. In both cases, the culture was stirred with T-line stirrer at 150 rpm. Microbial growth was determined by measuring absorbance at 650 nm. Viable cell population was estimated by plating samples on MRS agar. *Lactobacillus* LBL-4 cells were harvested from the medium samples by centrifugation (4000 × g at 4°C for 15 min). The addition of lysozyme (Sigma) (1 mg/mL at 40°C for 2 h) led to AP release. The pooled supernatant fluids (4000 × g at 4°C for 30 min) of four successive centrifugations gave the AP extracts, concentrated further by ultrafiltration on Amicon UM-10 membrane.

### 2.2. Hydrolysis

Solutions (6%) of casein and soybean isolate as well as 10% suspension of green algae *Scenedesmus* extracted by ethanol were used as substrates [28]. The enzymatic hydrolysis by subtilisin DY (5000 units/g substrate protein) was carried out at pH 7.8 and 50°C for 4 h [29]. After hydrolysis, the reaction mixtures were centrifuged at 2000 × g for 15 minute yielded in clear solutions (pH 7.0) containing 3.6% hydrolyzed protein with DH 20–22% (casein, soybean) and 19–20% (algae). Samples (100 mL) of protein hydrolysate solutions were incubated at 45°C with cell-free AP extract (36 AP units), and the additional hydrolysis *in vitro *was run for 10 h. Aliquots were taken, adjusted to pH 3.0 to obtain, clear solution, filtered, and applied for analysis. After the process was over (10 h), some samples of the reaction mixture were subjected to ultrafiltration (Amicon UM-10 membrane) to evaluate the enzyme recovery. In order to study the enzyme/substrate ratio, hydrolysis for 10 h of both casein and algae peptides were performed in the range of 5–20 AP units/g hydrolyzed protein. Industrial scale *Scenedesmus* algae protein hydrolysate [28] was extracted by water (1 : 9, w/v) to obtain clear solution (DH 19-20%; 5.5% hydrolyzed protein). Peptides were inoculated by 10% (v/v) culture of *Lactobacillus* LBL-4 grown in sterilized skim milk (10^7^-10^8^ cfu/mL) and fermented for 72 h at 45°C in order to follow the *in vivo* hydrolysis.

### 2.3. Analysis

Aminopeptidase activity of the cell-free LBL-4 extract was analyzed using L-Ieucine-*p*-nitroanilide (Serva) as substrate [30]. A unit of AP activity was defined as the amount of enzyme producing 1 *μ*mole *p*-nitroaniline per minute (E_410_ = 8800 M^−1^cm^−1^). Carboxypeptidase activity was measured by spectrophotometric method using N-carbobenzyloxy-L-Leu (Serva) as substrate. One unit of enzymatic activity was defined as the amount of enzyme that produced an increase in absorbance at 570 nm of 0.01 units [29]. Proteolytic activity of Subtilisin DY was defined as *μ*g tyrosine released per min during the hydrolysis of 1.2% casein (Fluka) at pH 7.4, 37°C [31]. 

The concentration of amino nitrogen was determined according to Adler-Nissen [32] and the DH was estimated as the percentage of the cleaved peptide bonds. The protein content was measured by Kjeldahl's method [33]. 

BioGel P-2 extra fine, from Biorad (USA) was packed in a column (13 × 140 mm) equilibrated by 0.1 M acetic acid containing 0.28% SDS as it was recommended earlier [34]. Samples (0.1 mL) were applied and eluted (velocity 18 mL/h) by starting buffer. Absorbance at 280 nm was measured continuously by a monitor UV-l, Pharmacia (Sweden). The following molecular weight markers were applied: Insulin B-chain (Sigma) -3495 Da; L-glutathione (oxidized) (Serva) -612 Da; L-glutathione (reduced) (Serva) -307 Da; Trp-Leu (Merck) -317 Da; Gly (Sigma) -75 Da. 

For sensory analysis, the peptide solutions (0.25 mL) were compared with 0.25 mL of standard quinine sulfate solutions (0.001–0.004%) for bitterness. The bitterness level was expressed in corresponding concentration of the quinine sulphate.

## 3. Results and Discussion

### 3.1. Aminopeptidase Sources

Yoghurt strain *Lactobacillus *LBL-4 was investigated as a considerable food-grade source of intracellular aminopeptidase using a crude cell extract, because the strain is isolated from traditional yogurt in Bulgaria. Further study regarding the isolation and characterization of the aminopeptidase will be published. 

**Figure 1:**
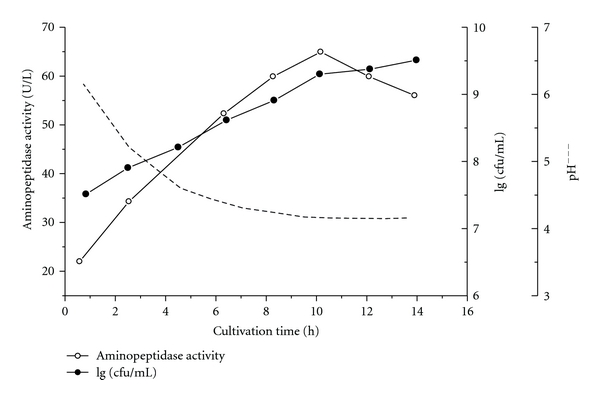
Biosynthesis of intracellular aminopeptidase activity during the *Lactobacillus* LBL-4 cultivation at natural pH decrease; (○): aminopeptidase activity; (●): 1g cfu/ml.

**Figure 2:**
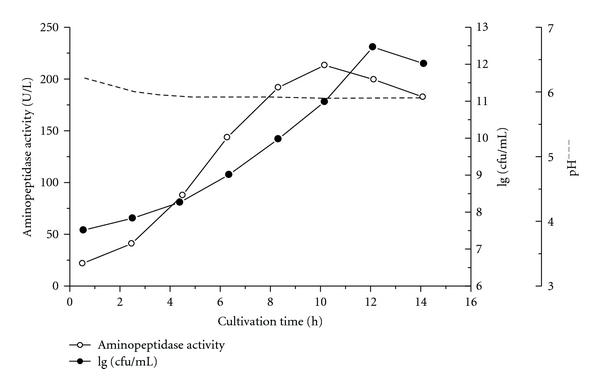
Biosynthesis of intracellular aminopeptidase activity during the *Lactobacillus* LBL-4 cultivation at maintained pH ~6.0; (○): aminopeptidase activity; (●): 1g cfu/ml.

Figures 1 and 2 represent the biosynthesis of intracellular AP activity during the cultivation of LBL-4 at maintained pH 5.8–6.0 as well as at natural pH decrease down to pH 4.0. It is clear that the number of living LBL-4 cells (cfu/mL) as well as their growth phase define the enzyme yield (Figure 2). Cultivation at constant pH for 12 h lead to maximal cell density of 5 × 10^12^ cfu/mL; however, maximal AP activity (>200 AP U/l medium) was yielded from logarithmic-phase cells harvested after 8–10 h fermentation time. The low AP activity (70 U/l medium) obtained in the case of lactic acid fermentation at maintained pH ~6.0 (Figure 2) underlined the importance of pH control during the cultivation process. The obtained extract can be subjected to evaporation in vacuum, and no activity reduction was observed using up to 45°C of the water bath. An ultrafiltration procedure can also be applied successfully. Neither endopeptidase nor carboxypeptidase activities were detected in the cell-free extract, which is evidence that the *in vitro *peptide hydrolysis was due to the action of the aminopeptidase only.

Exopeptidases from different sources have been successfully used for further hydrolysis of bitter peptides. The extracts from animal sources should be purified from the contaminants presented. Mycotoxins make microbial exopeptidases less suitable for food application [6]. The low content of exopeptidases in plant sources as well as laborious procedures for isolation appears to be a limitation for their practical use. 

It was discovered that baker's yeast residue autolysis demonstrating carboxypeptidase activity [9] is an attractive exopeptidase source. Cell wall *Saccharomyces* carboxypeptidase has been caused fate of peptides bitterness (200 g cell walls/kg peptides). According to our studies, the use of yeast residues can be accompanied by microbial contamination, so the presence of preservatives could be required during the hydrolytic process. 

The strain *Lactobacillus* LBL-4 is an attractive microbial source of AP from several points of view: (a) the culture is used for production of traditional food; (b) relatively high level of AP activity after short fermentation time, (c) after sterile filtration the hydrolysis can be carried out in aseptic conditions; (d) possibility for industrial-scaled cultivation.

### 3.2. Hydrolysis by Cell-Free Aminopeptidase

The enzymic protein hydrolysates used as model substrates for additional hydrolysis by AP activity were obtained after subtilisin-induced hydrolysis of casein, soybean isolate, as well as *Scenedesmus* cell protein as described before [28]. In all cases, the enzymatic hydrolysis proceeded to a limited DH (20–26%) although a proteolytic enzyme with broad specificity was used. This result could be explained by the formation of relatively stable polypeptide structures, containing in their interior some peptide bonds unsusceptible to the enzyme action [35]. On the other hand, it is known that casein hydrolysates (with high *Q* value) as well as soybean isolate hydrolysates with DH 20–26% manifest a strong bitterness [36]. 

The influence of enzyme/substrate ratio was studied using the initial protein hydrolysates from casein (DH 20–22%) and algae protein (18–20%). Algae peptides were additionally degraded by 5, 10, and 20 AP U/g hydrolyzed protein, obtaining final products with DH 32%, 38%, and 43% respectively. Subtilisin-digested casein incubated at the same enzyme/substrate ratio resulted in hydrolysates with DH 34%, 40%, and 46%. A range of 8–10 AP U/g hydrolyzed protein could be recommended for the debittering process combining an acceptable DH of the final products and enzyme save. The subtilisin activity prior to addition of the AP activity was not inactivated due to two reasons: (a) minimum chemical introduction in food process, and (b) the enzyme continues its action on the new peptides formed under the AP action.

**Figure 3:**
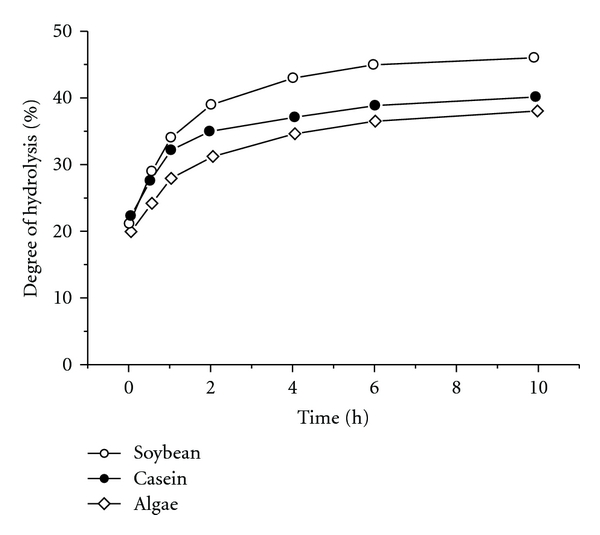
Increase of DH during the additional cleavage of protein hydrolysates from soybean (○), casein (●) and algae (*◊*) protein induced by 8 AP U/g peptides.

Figure 3 illustrates the additional cleavage of protein hydrolysates with different *Q* value by AP cell-free extract from LBL-4. A significant increase of DH during the first 4 h of additional hydrolysis process corresponded to a typical enzyme kinetic. After 10 h the process was ended, and the initial DH (20–22%) increased up to 40–42%, 46–48%, and 38–40% for the casein, soybean, and algae proteins, respectively. No significant differences in DH were observed at similar experiments carried out for up to 24 h hydrolysis. 

**Figure 4:**
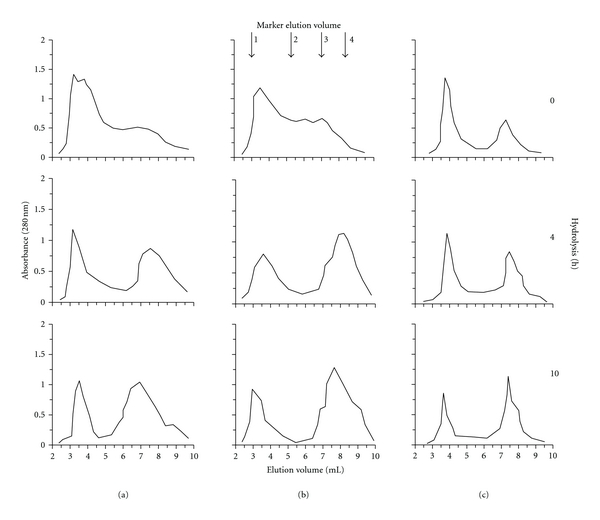
Gel chromatography on BioGel P-2 of protein hydrolysates from soybean (a), casein (b) and algae (c) obtained during the additional cleavage by 8 AP U/g peptides. MW markers were used as follows: (1) Insulin B chain (3495 Da); (2) L-glutation (oxidized) (612 Da); (3) Trp-Leu (317 Da); (4) Gly (75 Da).

The enzyme action was followed by gel chromatography (Figure 4) showing that considerable changes occurred during the hydrolysis in the period of 4 to 10 h when the DH increased gradually. The gel chromatography on BioGel P-2 was applied by addition of 0.28% SDS reducing the interactions of hydrophobic aromatic amino acids (resp. their short peptides) and matrix [34]. This gave us an opportunity to follow the reaction according to the molecular weights of the released peptides. Gel chromatography profiles demonstrates 3 main fractions: (i) relatively high-MW peptides (2000 Da and more); (ii) middle fraction consisting of 3–8 amino acid residues mainly; (iii) dipeptides and free amino acids. Practically, oligopeptides consisting of 2 to 8 amino acid residues appeared to be substrates for the aminopeptidase's action during the additional hydrolysis. After the end of hydrolysis, a reduction of the middle fraction can be observed in all cases, and the final hydrolysates obtained consist mainly of high MW fraction and free amino acids. The presence of high MW peptides in the hydrolysates is a positive factor balancing high osmolarity of the free amino acids. After the AP treatment, samples of the hydrolysates (casein, algae) were subjected to ultrafiltration showing 40–50% enzyme recovery with possibility for reuse.

The bitterness level of the initial soybean and casein hydrolysates (high *Q* value [1]) was near to the bitterness of 0.003-0.004% quinine sulphate. After the additional AP hydrolysis, the bitter taste reduced significantly and was comparable with 0.0005–0.001% quinine sulphate (near to the threshold). Even the debittering effect was very clear, the number of panelists (5 people) makes these results with preliminary character. The algae protein hydrolysate (low *Q* value [28]) manifesting a weak bitter taste was practically free of bitterness after the additional cleavage by AP. 

### 3.3. Hydrolysis during the *Lactobacillus* LBL-4 Fermentation

**Figure 5:**
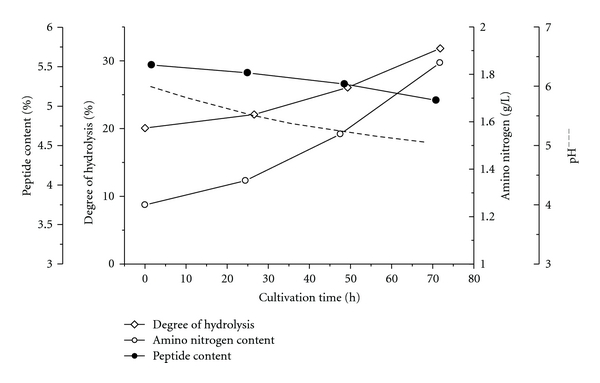
Additional hydrolysis of *Scenedesmus* protein hydrolysate during the *Lactobacillus* LBL-4 fermentation. (*◊*): degree of hydrolysis; (○): amino nitrogen content; (●): peptide content.

The results obtained during the hydrolysis *in vitro *by *Lactobacillus* LBL-4 AP challenged us to check the possibility for cleavage of the bitter peptides during the strain fermentation. The cultivation of *Lactobacillus* LBL-4 on algae protein hydrolysate—a model peptide system with low *Q* value— is followed on Figure 5. During the *in vivo* process, DH of algae hydrolysate increased to a more moderate level of 32%, while at the hydrolysis by cell-free AP, the DH reached 38% (Figure 3). The cultivation/hydrolysis time was much longer due to three reasons, mainly, (i) The AP content in the studied model system is approximately in an order less than in the hydrolysis experiments *in vitro*; (ii) the cell membrane limited the permeability of peptides; (iii) although the substrate solution has buffering capacity, pH value of the cultivation medium decreased gradually due to the released lactic acid. On the other hand, the presence of organic carboxylic acids is desirable due to their masking effect on the bitter taste [37].

During the fermentation process, the peptide content decreased (Figure 5) in the range of 10% from the initial value which was acceptable for a 72 h process. The final algae peptide solution was nonbitter; therefore, the additional hydrolysis *in vivo* could be recommended in the case of-low *Q* value proteins.

## 4. Conclusion

The strain* Lactobacillus *LBL-4 is an attractive microbial source of aminopeptidase with high potential for debittering of enzymatic protein hydrolysates, which could be successfully applied as food additives in medicine and sport. Both methods: the use of cell-free enzyme extract as well as cultivation of the strain on media containing hydrolyzed protein resulted in a significant reduction of bitterness.

## Abbreviations

AP^1^: Aminopeptidase
DH^2^: Degree of hydrolysis.
